# Effect of Zinc Foliar Fertilization Alone and Combined with Trehalose on Maize (*Zea mays* L.) Growth under the Drought

**DOI:** 10.3390/plants12132539

**Published:** 2023-07-03

**Authors:** Daniel Klofac, Jiri Antosovsky, Petr Skarpa

**Affiliations:** Department of Agrochemistry, Soil Science, Microbiology and Plant Nutrition, Faculty of AgriScience, Mendel University in Brno, Zemědělská 1, 61300 Brno, Czech Republic; xklofac@node.mendelu.cz (D.K.); jiri.antosovsky@mendelu.cz (J.A.)

**Keywords:** zinc fertilization, trehalose solution, maize, chlorophyll content, chlorophyll fluorescence parameters, root size, biomass production

## Abstract

Maize (*Zea mays* L.) is one of the most widely grown cereals in the world. Its cultivation is affected by abiotic stress caused by climate change, in particular, drought. Zinc (Zn) supplied by foliar nutrition can increase plant resistance to water stress by enhancing physiological and enzymatic antioxidant defence mechanisms. One of the possibilities to reduce the effect of drought on plant production is also the utilization of trehalose. In order to confirm the effect of the foliar application of selected forms of Zn (0.1% *w/v* solution)—zinc oxide micro- (ZnO) and nanoparticles (ZnO^NP^), zinc sulphate (ZnSO_4_) and zinc chelate (ZnEDTA)—a pot experiment in controlled conditions was conducted in combination with trehalose (1% *w/v* solution) on selected growth parameters of maize exposed to the drought stress. A significant effect of coapplication of Zn and trehalose on chlorophyll content, chlorophyll fluorescence parameters, root electrical capacity, weight of maize aboveground biomass (AGB) and Zn content in AGB was found. At the same time, the hypothesis of a positive effect of carbohydrates on increasing the uptake of foliar-applied Zn was confirmed, especially for the ZnEDTA and ZnSO_4_. This paper presents the first empirical evidence of the trehalose addition to sprays for zinc foliar fertilization of maize proving to be an effective way of increasing the resistance of maize grown under drought stress conditions.

## 1. Introduction

Drought is one of the main factors limiting crop yields in many parts of the world. Forecasts of decreasing rainfall and increasing evaporation in maize-growing areas will further exacerbate production losses [1]. Drought has a significant impact on plant production and plant nutritional status [2]. On the contrary, an adequate and balanced supply of mineral nutrients for crops is an integral part of combating the effects of drought, and the mineral nutrient status of plants plays a crucial role in increasing plant resistance to drought stress [3].

Zinc (Zn) is an essential micronutrient that plays fundamental roles in crop resistance against the drought stress by regulating various physiological and molecular mechanisms [4]. Although most soils contain sufficient amounts of zinc, its uptake can be limited due to a number of chemical and physicochemical conditions in the soil [5]. However, zinc is the most widely deficient trace element in agriculture [6], and its deficiency causes a reduction in crop yield of up to 40% [7]. Zinc deficiencies often occur on alkaline soils, light sandy soils, or soils with relatively high levels of available phosphorus [8]. Maize (*Zea mays* L.) is known as a crop with high zinc requirements compared to other micronutrients [9], with optimum levels of zinc in maize tissue ranging from 20 to 60 mg/kg [10]. Foliar application of zinc can be used as a supplement to maize nutrition, especially in situations where soil properties may reduce the bioavailability of Zn to plants. The effect of foliar fertilization of maize with zinc is presented in several studies. For example, the problem of zinc deficiency was addressed by Anees et al. [11], who foliarly applied a 0.1% zinc solution in a small-plot experiment with maize and observed increases in grain yield, 1000 grains weight, number of grains per ear and plant height. González-Cavallo et al. [12] found an increase in maize plant height and dry matter (DM) weight after zinc fertilization in the sulphate form. The sulphate form of zinc was also foliarly applied to maize by Imran et al. [13], resulting in an increase in grain yield, grain weight and grain Zn content. Subbaiah et al. [9] demonstrated a significant effect of ZnO application in nanoparticle size (NPs) on maize grain yield and grain zinc content. Selected forms of zinc (sulphate, oxide and oxide in nano size) were applied to maize in pots and in a small-plot field experiment by Umar et al. [14]. In pot experiments, zinc application always resulted in a positive effect on plant height and dry matter weight, with the highest values provided by ZnO-NPs treatment. In the small-plot field experiment, application of every examined form of zinc also had a positive effect on grain yield, 1000 grains weight and plant height, again with the highest values provide by application of ZnO-NPs.

One of the possibilities to reduce the effect of drought on plant production is to use trehalose [15]. Trehalose is a naturally occurring nonreducing disaccharide formed by two molecules of D-glucose linked by an α,α-1,1-glycosidic bond [16]. It occurs naturally in species such as *Myrothamnus flabellifolius* or *Selaginella lepidophylla*, known as resurrection plants, which have the ability to survive long periods of drought and dehydration. The trehalose content of these plants can be as low as one percent by dry matter weight [17,18]. Trehalose in the plant acts as a source of energy and, above all, as a protector against dehydration and frost [19]. In the drought period, it forms a hydrogen bond with cell membranes in place of water molecules to help maintain their stability [20,21]. When strongly dehydrated, it vitrifies into a glassy form with the ability to protect biomolecules and preserve their functions until subsequent hydration [22]. Exogenous application of trehalose has been a widely researched topic on various crops in recent years, with positive results. For example, Akram et al. [23] achieved an increase in aboveground biomass (AGB) weight and chlorophyll content by foliar application of trehalose to radish (*Raphanus sativus* L.). Alam et al. [24] exogenously supplied trehalose to seedlings of three species of the *Brassicaceae* family and monitored their development under drought conditions. The treated seedlings of all species showed an increase in biomass weight, chlorophyll content, glutathione content and a decrease in the content of stress markers H_2_O_2_ and malondialdehyde compared to the treatments without trehalose. Trehalose was applied foliarly to maize plants grown under the drought conditions by Ali et al. [25]. Compared to the untreated control, trehalose increased biomass production, improved photosynthetic attributes and activity of some key antioxidant enzymes such as peroxidase (POD) or catalase (CAT). Ibrahim et al. [26] foliarly applied trehalose to wheat (*Triticum aestivum* L.) plants grown under the drought conditions. The treatments with trehalose showed improved growth parameters compared to the untreated control, such as AGB, plant height and leaf area size. The application of trehalose also had a positive effect on the content of flavonoids and phenolic compounds important for the scavenging of reactive oxygen species. Seed drenching and foliar application of trehalose were tested under the drought conditions by Shafiq et al. [27] on *Raphanus sativus* L. plants. Both treatments of trehalose application increased root fresh weight, phenolic content, glycinebetaine, superoxide dismutase (SOD), CAT and POD activity. Basil (*Ocimum basilicum* L.) plants were used in an experiment by Zulfiqar et al. [28]. They described a reduced impact of drought on plants by foliar application of trehalose solution and observed an increase in AGB weight, plant length, chlorophyll content and higher CO_2_ assimilation. They also observed a decrease in the stress markers H_2_O_2_ and malondialdehyde and an increase in the activity of CAT, SOD and POD.

**Figure 1:**
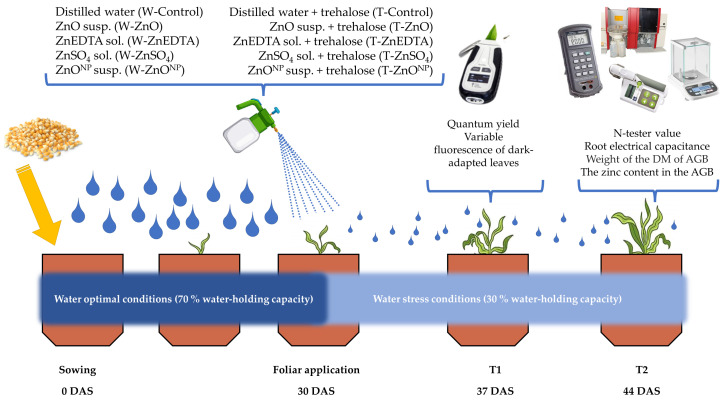
Timetable of the greenhouse pot experiment. DAS: day after sowing.

This study aimed to investigate the effect of foliar application of trehalose in combination with zinc applied in selected forms: zinc oxide micro- (ZnO) and nanoparticles (ZnO^NP^), zinc sulphate (ZnSO_4_) and zinc chelate (ZnEDTA), on the selected parameters of maize grown under the drought conditions (Figure 1). The pot experiment verifies the hypothesis that zinc application in combination with trehalose will increase the drought resistance of maize plants.

## 2. Results

### 2.1. N-Tester Value and Zinc Content in Plant

**Figure 2:**
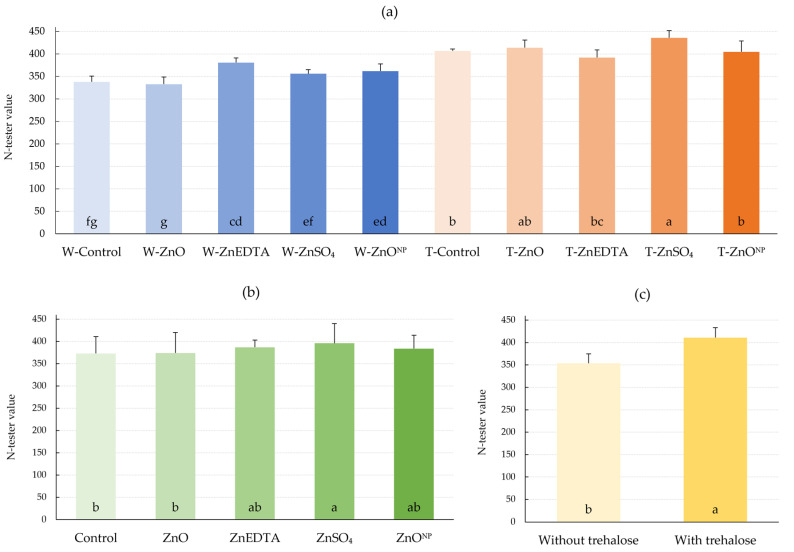
Effect of foliar fertilization on N-tester values. (**a**) Effect of treatments on N-tester value; (**b**) effect of zinc form on N-tester value; (**c**) effect of trehalose on N-tester value. The mean values sharing the same letter are not significantly different from each other (*p* ≤ 0.05) according to the Fisher test (each factor was evaluated separately). Error bars represent the standard deviation of the mean.

N-tester values, reflecting chlorophyll content, were significantly increased in plants fertilized with zinc chelate applied to plants in water (W) spray (W-ZnEDTA) and nano zinc oxide (W-ZnO^NP^) (Figure 2). Compared to the W-Control, N-tester values increased by 12.7% and 7.1%, respectively. In the case of coapplication of zinc with trehalose (T), there was a significant increase in every examined treatment, and even the application of trehalose alone without zinc significantly increased the N-tester value by 20.4%. The application of zinc without including the effect of trehalose resulted in increased N-tester values; especially, ZnSO_4_ significantly increased the N-tester value by 6.2%. The average effect of trehalose was also significant with the 16.1% increase in the observed parameter.

**Figure 3:**
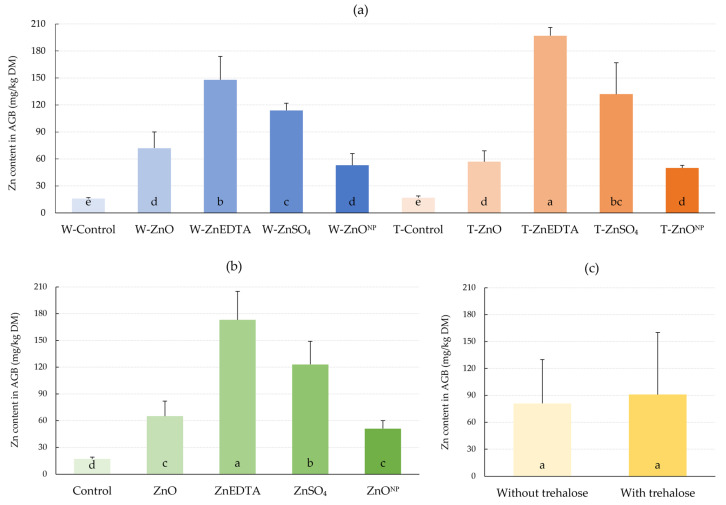
Effect of foliar fertilization on Zn content in dry matter of maize plants. (**a**) Effect of treatments on Zn content; (**b**) effect of zinc form on Zn content; (**c**) effect of trehalose on Zn content. The mean values sharing the same letter are not significantly different from each other (*p* ≤ 0.05) according to the Fisher test (each factor was evaluated separately). Error bars represent the standard deviation of the mean. AGB: aboveground biomass; DM: dry matter.

The effect of zinc foliar application on Zn content in DM of maize AGB, expressed as a mean for each of the Zn forms, was as follows: ZnEDTA (156 mg/kg increase over the control) > ZnSO_4_ (106 mg/kg) > ZnO (48 mg/kg) > ZnO^NP^ (34 mg/kg) (Figure 3). The addition of trehalose resulted in a significant difference of 33.1% in zinc content in maize DM for the ZnEDTA (T-ZnEDTA) treatment compared to the ZnEDTA treatment without trehalose (W-ZnEDTA). A nonsignificant increase by 15.8% occurred after coapplication of trehalose with ZnSO_4_. There was a nonsignificant decrease by 20.8% and 5.7% in the treatments with trehalose ZnO (T-ZnO) and ZnO^NP^ (T-ZnO^NP^) compared to the treatments without saccharide (W-ZnO and W-ZnO^NP^), respectively. The application of trehalose without zinc resulted in a relatively increased Zn content in plant AGB by 12.3%; the difference was, however, not significant.

### 2.2. Chlorophyll Fluorescence Parameters

**Figure 4:**
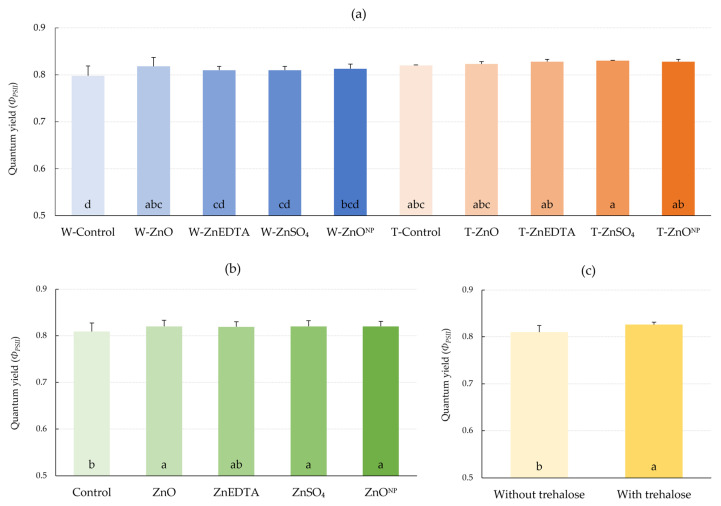
Effect of fertilization on quantum yield (*Φ_PSII_*). (**a**) Effect of treatments on *Φ_PSII_*; (**b**) effect of zinc form on *Φ_PSII_*; (**c**) effect of trehalose on *Φ_PSII_*. The mean values sharing the same letter are not significantly different from each other (*p* ≤ 0.05) according to the Fisher test (each factor was evaluated separately). Error bars represent the standard deviation of the mean.

A significant increase in quantum yield by 2.5% was measured for the ZnO form without trehalose (W-ZnO) in comparison with the control (Figure 4). In the case of combined application with trehalose, there was a statistically significant increase in quantum yield of photosystem II (*Φ_PSII_*) for every T-treatment. The highest increase was measured for the T-ZnSO_4_ form, by 4.0% compared to the control. The effect of trehalose treatments on quantum yield was significant.

**Figure 5:**
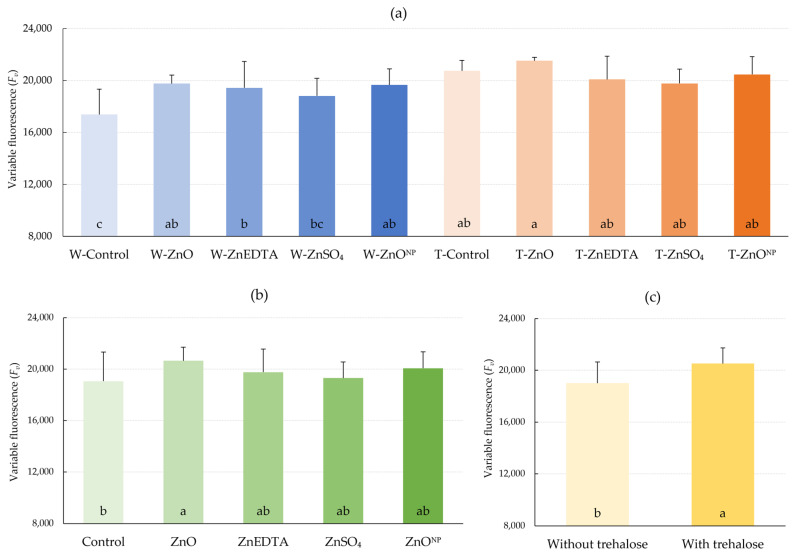
Effect of fertilization on variable fluorescence of the dark-adapted leaves (*F_v_*). (**a**) Effect of treatments on *F_v_*; (**b**) effect of zinc form on *F_v_*; (**c**) effect of trehalose on *F_v_*. The mean values sharing the same letter are not significantly different from each other (*p* ≤ 0.05) according to the Fisher test (each factor was evaluated separately). Error bars represent the standard deviation of the mean.

Variable fluorescence of the dark-adapted leaves (*F_v_*), which explains (describes) the ability of photosystem II (*PSII*) to absorb radiation [29], increased significantly for every treatment of zinc and its combination with trehalose compared to the W-Control (Figure 5a), except for the W-ZnSO_4_. According to Figure 5c, the effect of trehalose without considering the effect of zinc was significant by 7.9%. The highest values of *F_v_* were measured for the treatments with zinc application in ZnO form (W- and T-ZnO) compared to other Zn forms.

### 2.3. Root Electrical Capacitance (C_R_)

**Figure 6:**
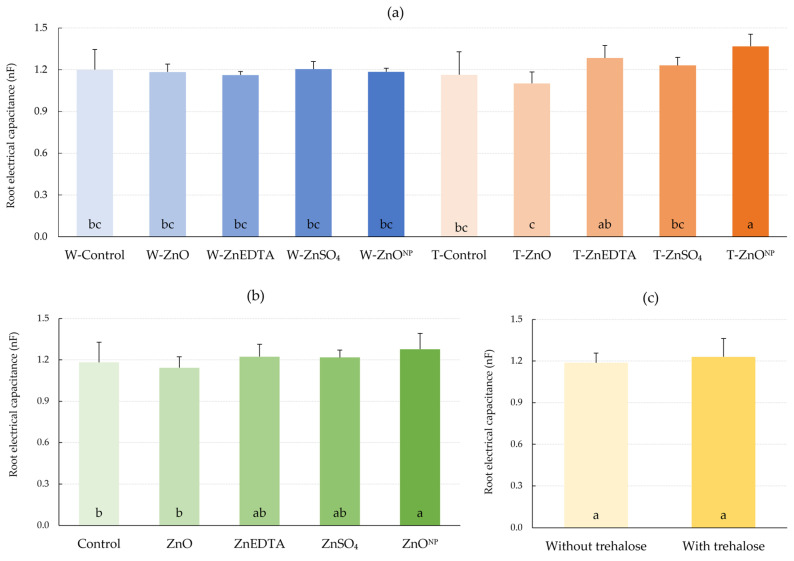
Effect of foliar fertilization on root electrical capacitance (*C_R_*). (**a**) Effect of treatments on *C_R_*; (**b**) effect of zinc form on *C_R_*; (**c**) effect of trehalose on *C_R_*. The mean values sharing the same letter are not significantly different from each other (*p* ≤ 0.05) according to the Fisher test (each factor was evaluated separately). Error bars represent the standard deviation of the mean.

Although root electrical capacitance is an indirect method of determining root system size, a number of studies have shown a positive relationship between root electrical capacitance and root length and weight [30,31,32] The effect of trehalose on root electrical capacitance regardless of the Zn form was 3.6% higher according to Figure 6c, but the difference was not statistically significant. The comparison of zinc forms regardless of trehalose application is described in Figure 6b. According to these results, the effect of ZnO^NP^ application was significant compared to the control by 8.0% (Figure 6b). The positive effect of zinc in the treatments without trehalose was not evident, and all values of *C_R_* showed a decrease except W-ZnSO_4_, where no change from the W-Control was observed. In combination with trehalose, zinc application had a positive effect on root size in the T-ZnEDTA and T-ZnSO_4_ treatments. The increase in *C_R_* was 7.0% and 2.6%, respectively. The significantly highest value of root electrical capacitance was determined after the treatment with T-ZnO^NP^. The increase was 14.1% higher compared to the W-Control (Figure 6a).

### 2.4. The Weight of Dry Matter of Maize Aboveground Biomass

**Figure 7:**
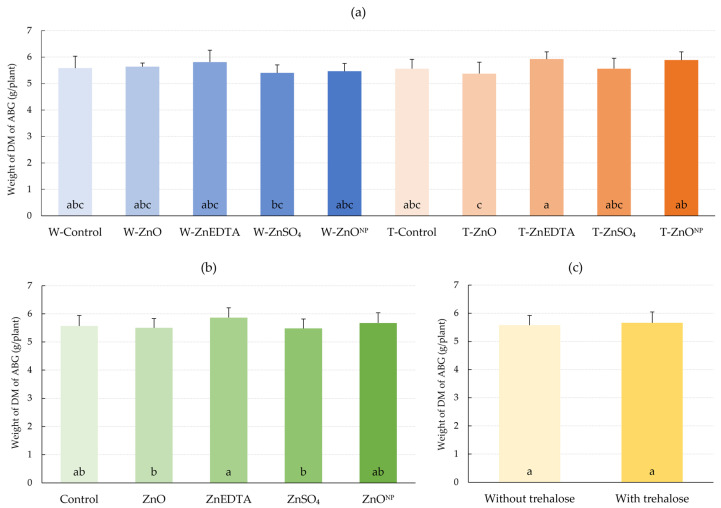
Effect of foliar fertilization on DM weight of AGB. (**a**) Effect of treatments on DM weight; (**b**) effect of zinc form on DM weight; (**c**) effect of trehalose on DM weight. The mean values sharing the same letter are not significantly different from each other (*p* ≤ 0.05) according to the Fisher test (each factor was evaluated separately). Error bars represent the standard deviation of the mean. AGB: aboveground biomass; DM: dry matter.

The effect of foliar application of zinc regardless of trehalose application on AGB production of maize plants was not significant (Figure 7b). The relatively highest DM production of AGB was found in the ZnEDTA, compared to the control by 5.3%. Application of ZnSO_4_, ZnO and ZnO^NP^ did not produce any statistical effect. The effect of trehalose regardless of Zn form on DM weight was not significant (Figure 7c), but there was a relative increase of 1.5%. The highest effect of combined application of trehalose and zinc was observed in comparison with the control after treatment with the T-ZnEDTA (by 6.3%), and T-ZnO^NP^ (by 5.6%) (Figure 7a). The differences were, however, insignificant.

## 3. Discussion

### 3.1. N-Tester Value and Zn Content in Plant

Foliar application of every form of zinc had a positive effect on relative N-tester values with the highest significant increase in the ZnSO_4_ form of 6.2% compared to the control (Figure 2). Without the addition of trehalose, the W-ZnEDTA application resulted in the highest chlorophyll content (12.7% increase over the control), while the sulphate form in combination with trehalose resulted in an increase of 29.0%. Only the ZnO treatment showed a nonsignificant decrease of 1.5% in chlorophyll content compared to the control. The study by Heitholt et al. [33] reported a significant increase in chlorophyll content values in soybean plants fertilized with zinc to the soil. The positive effect of zinc fertilization on nitrogen metabolism was also confirmed by Mosaad et al. [34], where the addition of 3.64 kg of Zn in sulphate form (16 kg ZnSO_4_.7H_2_O) to soil increased nitrogen efficiency in rice by 12.3%.

The measured N-tester values, expressing the chlorophyll content of trehalose-fertilized maize (T-Control), were significantly increased compared to the W-Control. The addition of trehalose to the spray significantly increased the chlorophyll content by 20.4%. The corresponding results are presented by Akram et al. [23], in whose study foliar application of trehalose increased the plant nitrogen content of three *Brassica* species. The positive effect of exogenous application of carbohydrates, and hence trehalose, can be explained on the basis of the research findings of Hess [35]. In his study, application of 0.5% glucose to the growth medium increased nitrate reductase activity in *Arabidopsis thaliana* roots. The increased activity was due to an increase in nitrogen content in the ammonium form and its subsequent incorporation into amino acids in the GS-GOGAT cycle [36] necessary for chlorophyll formation [37]. Sadak [38] observed an increase in chlorophyll a + b content by 31.4% and 52.4% in drought-stressed *Trigonella foenum-graecum* plants after foliar application of a solution containing 250 and 500 µM trehalose, respectively. Also, Zhou et al. [39] observed an 11.7% increase in GS-GOGAT activity due to sucrose application in lettuce plants.

The zinc content of maize plants was increased by foliar application in the following order: ZnEDTA > ZnSO_4_ > ZnO > ZnO^NP^ > Control without Zn application (Figure 3). This trend is consistent with the results of Alexander and Hunsche [40], who compared the penetration rates of different forms of zinc through the cuticle of tomato. The explanation for the different effect of Zn forms on zinc content and subsequent chlorophyll is the ability of zinc to penetrate through the cuticle. This is determined by several factors, including their water permeability. Aqueous solubility of a foliar nutrient compound is an important factor determining foliar penetration of the nutrient concerned, as the absorption of nutrient ions only occurs when the nutrient chemical is dissolved in the aqueous phase on a leaf surface [41]. The forms of zinc we used can be grouped into two categories based on the solubility in water: soluble salts and chelates (ZnSO_4_, and ZnEDTA) and sparingly soluble Zn compounds (ZnO, and ZnO^NP^). While ZnSO_4_, due to its high solubility and low cost, is the most widely used inorganic Zn source for foliar application, zinc chelates (e.g., ZnEDTA) are more effective in foliar application but are considerably more expensive than inorganic compounds [42]. The addition of trehalose to the zinc solution increased the amount of total zinc in three of the four treatments in maize biomass, which is supported by the work of Xia [43], who observed an increase in total Zn content when sucrose and ZnSO_4_ were applied together. This synergistic effect can be explained in three ways: (1) longer drying time of the solution on the leaf; (2) better penetration through the cuticle; (3) better rate of Zn translocation from the absorption site to other parts of the plant [44]. Fernández et al. [45] reported the possibility of reducing the spray deliquescence relative humidity by using additives, which may be trehalose, which will increase the rate of leaf penetration.

### 3.2. Chlorophyll Fluorescence Parameters

The relative increase in quantum yield was observed in every treatment compared to the control (Figure 4). A significant increase occurred after every treatment with trehalose application (T-) and after the foliar spray of ZnO without trehalose (W-ZnO). The importance of zinc for quantum yield was described, for example, by Roosta et al. [46], where zinc deficiency in lettuce nutrition caused a significant decrease in *Φ_PSII_*. Under drought conditions, Pilon-Smits et al. [47] achieved a statistically significant increase in *Φ_PSII_* of transgenic tobacco capable of metabolizing trehalose compared to wild-type tobacco (as they refer to it), which is consistent with our observations. Since the quantum yield value is dependent on chlorophyll content, its increase can be explained by the increase in nitrogen assimilation in the plant required to increase chlorophyll content [38].

External stress, such as that caused by drought, is one of the main factors that damages thylakoids and thus reduces variable fluorescence [48]. This suggests a positive effect of zinc application, both solely or in combination with trehalose, on reducing thylakoid damage and consequently preventing the reduction of *PSII* activity. Our experiment confirmed the positive effect of both zinc and trehalose on variable fluorescence, where the increase was significant for every treatment except ZnSO_4_ (Figure 5). A positive effect of lower zinc dose on chlorophyll amount and subsequent fluorescence parameters in tomato was observed by Cherif et al. [49]. Under inadequate zinc nutrition, Roosta et al. [46] observed a statistically significant decrease in variable fluorescence in lettuce. Pilon-Smits et al. [47] described a statistically significant increase in *F_v_* under drought conditions in transgenic tobacco capable of producing trehalose.

### 3.3. Root Capacity

Several studies have shown the positive effect of soil fertilization with zinc on root growth [50,51]. However, due to the low mobility of Zn in the phloem after foliar application [52], an increase in root system cannot be assumed, and this is confirmed by the observed trend where foliar application of zinc alone did not produce any significant effect on root capacity. However, a relative increase was observed for the T-ZnEDTA, T-ZnSO_4_ and T-ZnO^NP^ treatments, while the *C_R_* increase was significant for T-ZnO^NP^.

### 3.4. Dry Matter Weight

No significant increase in dry matter weight of AGB was observed after zinc and trehalose treatments. The highest relative increases were in the ZnEDTA and T-ZnEDTA treatments by 4.2% and 6.2%, respectively. Similarly, the inconclusive effects of foliar zinc fertilization on biomass production [53,54] are confirmed by our presented results. Wang et al. [55] found that the foliar Zn application did not significantly affect the biomass and grain yield of maize and wheat. These results contradict the findings of the study of Umar et al. [14], where ZnO and nano ZnO application increased DM weight by 6.9% and 52.8%, respectively. Application of the sulphate form of Zn increased DM weight by 37.5%. Xia et al. [56] applied sucrose along with ZnSO_4_ at different nitrogen doses, resulting in a DM weight reduction from 10.2% to 1.7% depending on the nitrogen dose. Foliar application of trehalose to *Trigonella foenum-graecum* with deficit irrigation was studied by Sadak [38], who achieved an increase in DM weight by 47.2% and 62.5% by applying solutions with concentrations of 250 and 500 µM trehalose, respectively. An experiment was conducted with foliar application of trehalose on wheat by Ibrahim and Abdellatif [26]. They reported an increase in DM weight of 49.1% and 130.4% at 10 days and 20 days interval irrigation, respectively.

## 4. Materials and Methods

The effect of foliar application of zinc, trehalose, and their combinations on the growth parameters of maize was studied under the conditions of a pot greenhouse experiment conducted at Mendel University in Brno (Brno, Czech Republic).

### 4.1. Materials and Experimental Design

The SY ORPHEOUS maize hybrid (Syngenta Czech s.r.o., Prague, Czech Republic) was used in the experiment as a model crop. The experiment was established under controlled greenhouse conditions with controlled day/night length (12 h/12 h), temperature (25 °C/17 °C) and humidity (60%/80%). Plants were grown in 2.4 L plastic pots (height 17 cm, diameter 15 cm) with 2000 g of soil. The physicochemical properties of the soil used in the experiment are given in Table 1. The zinc content of the soil used was low [57].

Six seeds of maize were sown in each pot, and after 2 weeks, the number of plants was manually reduced to 4 plants per pot. Foliar applications of selected forms of zinc, trehalose and their mixtures were performed at the three-leaf stage. A list of the treatments performed is given in Table 2.

Pure chemicals and commercially available fertilizer were used as sources of zinc for the preparation of solutions. The source of the microparticle oxide form of zinc (ZnO) was zinc oxide (CAS 1314-13-2, purum p.a. 99.9% Sigma-Aldrich, St. Louis, MO, USA). Ethylenediaminetetraacetic acid disodium zinc salt tetrahydrate in the commercial preparation Lister Zn (CAS 14025-21-9, 15% Zn, Arkop Ltd., Bukowno, Poland) was used as the source of the chelated form of zinc (ZnEDTA). Zinc sulphate heptahydrate (CAS 7446-20-0, purum p.a, 99.0%, Sigma-Aldrich, St. Louis, MO, USA) was the source of the sulphate form of zinc (ZnSO_4_) and for nanoparticle zinc oxide, ZnO^NP^ (CAS 1314-13-2, ZnO nanoparticles aqueous dispersion, 20 wt %, 30–40 nm, US Research Nanomaterials Inc., Houston, TX, USA). The nano-sized zinc oxide suspension was sonicated in an ultrasonic bath before application (Sonorex Digital 10 P Sonicator, Bandelin Electronic GmbH, Berlin, Germany) to prevent particle agglomeration.

Plants were treated with the prepared solutions/suspensions at a rate of a 4 mL spray per pot (1 mL/plant). The spray was applied with a pressurized hand pump sprayer (DPZ 1500, ProGlass, Weilheim an der Teck, Germany). After foliar application, watering was reduced. The water stress levels, i.e., 30% water-holding capacity, were maintained on a gravimetric basis [61].

### 4.2. Measurement of Selected Parameters of Photosynthesis and Plant Growth in Maize

Seven days after the foliar application (T1), chlorophyll fluorescence parameters (quantum yield of photosystem II (*Φ_PSII_*)) and variable fluorescence of dark-adapted leaves (*F_v_*) were measured. Fourteen days after foliar application (T2), chlorophyll content (expressed as N-tester value), root electrical capacity (*C_R_*), weight of dry matter (DM) of aboveground plant biomass (AGB) and zinc content of AGB were determined.

#### 4.2.1. Chlorophyll Content (N-Tester Value)

The relative chlorophyll content was determined using a handheld Yara N-tester (Yara International ASA, Oslo, Norway). The measurement principle is based on the transmittance of the leaves for wavelengths 650 nm (red) and 940 nm (NIR) [62]. By measuring both wavelengths together, it is possible to correct the data for any differences due to different leaf thicknesses. Based on the red and NIR colour values, the N-tester calculates a numerical dimensionless value that is proportional to the amount of the total chlorophyll present in the leaf [63]. The chlorophyll content was measured at the center of the leaf blade of the youngest fully developed leaf, where each measured value corresponds to the average of 30 measurements per plant. For each experimental treatment, 3 plants (*n* = 90) were measured.

#### 4.2.2. Chlorophyll Fluorescence Parameters

Chlorophyll fluorescence determination was performed with a PAR-FluorPen FP 110-LM/S fluorometer (Photon Systems Instruments, Drásov, Czech Republic). The FluorPen 1.1 software was used to analyse the measured data. The method is able to estimate the photosynthetic output expressed as energy flux through photosystem II (*PSII*) [64,65]. Before measuring chlorophyll fluorescence, plants (centre of the leaf blade of the youngest fully developed leaf) were adapted in the dark for 1 h. For each experimental treatment, 16 plants (*n* = 16) were measured. The following parameters were determined: variable fluorescence of the dark-adapted leaves (*F_v_*), which expresses the ability of the photosystem II to absorb radiation, from the *F_m_*-*F*_0_ relationship (*F*_0_—minimal fluorescence from the dark-adapted leaves, *F_m_*—maximal fluorescence from the dark-adapted leaves) [66], and quantum yield of photosystem II (*Φ_PSII_*) as equivalent to *F_v_*/*F_m_*, which expresses the efficiency of *PSII* [67].

#### 4.2.3. Root Electrical Capacitance

At the time of sampling (T2), the pots were flooded with water to ensure 100% water-holding capacity. Subsequently, root electrical capacitance was measured using a VOLTCRAFT LCR 4080 handheld instrument (Conrad Electronic GmbH, Wels, Austria). Root electrical capacitance (*C_R_*) was measured using a 1 kHz signal in nanofarads (nF). The first cathode (20 cm length, stainless steel) was placed in the soil to a depth of 10 cm. The second electrode (aluminium clamp) was fixed to the plant 2 cm above the soil surface. For each experimental treatment, 16 plants were measured (*n* = 16).

#### 4.2.4. Dry Weight of Aboveground Plant Biomass, Plant Zinc Content

Immediately after *C_R_* determination, plant aboveground biomass was collected. Aboveground corn plant mass was washed in 0.01 M HCl (for 3 min) to remove unabsorbed zinc from the leaf. Plants were then dried at 60 °C (Venticell 222 ECO line, MMM Medcenter Einrichtungen GmbH, Planegg, Germany) to constant weight followed by dry matter determination. The zinc content of the aerial plant matter was determined by atomic absorption spectrometry on a ContrAA 700 instrument (Analytik Jena AG, Jena, Germany) after prior grinding of the plants (Grindomix GM200, Retsch GmbH, Haan, Germany), their homogenization and microwave digestion on an ETHOS 1 instrument (Milestone Srl, Sorisole, Italy).

### 4.3. Statistical Data Processing

The effect of examined treatments on the monitored parameters was statistically evaluated in a pot experiment. The effect of different combinations of selected forms of zinc applied in water (W) and trehalose (T) solution was evaluated. The effect of selected forms of zinc (without taking trehalose into account) and the effect of trehalose (without taking zinc into account) were also evaluated. Statistical evaluation was performed by the Statistica 14 CZ software [68] (TIBCO Software, San Jose, CA, USA). The relationship between the treatments’ two-way analysis of variance (ANOVA) and follow-up tests according to Fisher (LSD) at the 95% (*p* < 0.05) level of significance were used, and homogeneity and normality of variances were tested by Levene’s and Shapiro–Wilk’s tests, respectively (*p* ≤ 0.05 level). The results were expressed as the mean ± standard deviation (SD).

## 5. Conclusions

Most of the current work deals with the separate application of carbohydrates or zinc. This work has provided the first insights into the coapplication of trehalose and different forms of zinc commonly used in agricultural practice or, in the future, used in the sense of ZnO nano. Conclusions based on this research show a clear positive effect of the synergistic action of both compounds on chlorophyll content (N-tester value), chlorophyll fluorescence parameters, root system and above ground biomass weight. At the same time, the hypothesis of a positive effect of carbohydrates on increasing the acceptability of foliar applied zinc was confirmed, especially for the chelated and sulphated forms. Zinc application either with or without trehalose also had a positive effect on the examined parameters, especially for quantum yield and variable fluorescence. The chelate form of zinc was found to be the most acceptable form of zinc for maize, followed by the sulphate and oxide forms. It is necessary to bear in mind, however, if the recommended practices are used in field production, the spatial specificity of the areas, due to soil and weather/climate variability, may challenge the previous conclusions.

Following these conclusions, further research should focus on verifying the effect of zinc fertilization in combination with trehalose under field conditions.

## Figures and Tables

**Table 1:** Physicochemical composition of the soil used in the experiment.

Soil Parameter	Value	Ref.
pH (CaCl_2_)	6.09	[58]
C_ox_	0.80%	[59]
Clay	20%	[60]
Slit	27%	
Sand	53%	
Cation Exchange Capacity	164 mmol/kg	[58]
N total	0.19%	
N-NH_4_^⁺^ (K_2_SO_4_)	1.48 mg/kg	
N-NO_3_^−^ (K_2_SO_4_)	17.2 mg/kg	
P (Mehlich 3)	36.4 mg/kg	
K (Mehlich 3)	400 mg/kg	
Ca (Mehlich 3)	2720 mg/kg	
Mg (Mehlich 3)	214 mg/kg	
Zn (Mehlich 3)	1.8 mg/kg	

**Table 2:** Design of treatments.

Treatment	Form of Zinc	Foliar Spray	Zn Concentration (*w*/*v*)	Trehalose Concentration (*w*/*v*)
Control	-	Distilled water (W)	0	0
W-ZnO	ZnO		0.1%	0
W-ZnEDTA	Zn-EDTA		0.1%	0
W-ZnSO_4_	ZnSO_4_		0.1%	0
W-ZnO^NP^	nano ZnO		0.1%	0
T-Control	-	Distilled water + trehalose (T)	-	1%
T-ZnO	ZnO		0.1%	1%
T-ZnEDTA	Zn-EDTA		0.1%	1%
T-ZnSO_4_	ZnSO_4_		0.1%	1%
T-ZnO^NP^	nano ZnO		0.1%	1%

W: foliar spray—water, T: foliar spray—water solution of trehalose.

## Data Availability

Not applicable.
